# Harnessing the power of botanical gardens: Evaluating the costs and resources needed for exceptional plant conservation

**DOI:** 10.1002/aps3.11495

**Published:** 2022-10-06

**Authors:** Megan Philpott, Valerie C. Pence, Burgund Bassüner, Ashley S. Clayton, Emily E. D. Coffey, Jason L. Downing, Christine E. Edwards, Raquel Folgado, Jason J. Ligon, Christy Powell, Joseph Francis Ree, Alexandra E. Seglias, Nellie Sugii, Peter J. Zale, Jacob Zeldin

**Affiliations:** ^1^ Center for Conservation and Research of Endangered Wildlife Cincinnati Zoo & Botanical Garden Cincinnati Ohio USA; ^2^ Center for Conservation and Sustainable Development Missouri Botanical Garden St. Louis Missouri USA; ^3^ Longwood Gardens Kennett Square Pennsylvania USA; ^4^ Southeastern Center for Conservation Atlanta Botanical Garden Atlanta Georgia USA; ^5^ Fairchild Tropical Botanic Garden Miami Florida USA; ^6^ The Huntington Library, Art Museum, and Botanical Gardens San Marino California USA; ^7^ San Diego Zoo Wildlife Alliance San Diego California USA; ^8^ Department of Research and Conservation Denver Botanic Gardens Denver Colorado USA; ^9^ Hawaiian Rare Plant Program, Harold L. Lyon Arboretum Honolulu Hawai'i USA; ^10^ Chicago Botanic Garden Glencoe Illinois USA

**Keywords:** botanic garden, collaboration, cryopreservation, ex situ, exceptional plant, micropropagation

## Abstract

**Premise:**

The effective ex situ conservation of exceptional plants, whether in living collections or cryo‐collections, requires more resources than the conservation of other species. Because of their expertise with rare plants, botanical gardens are well positioned to lead this effort, but a well‐developed strategy requires a clear understanding of the resources needed.

**Methods:**

Grant funding was obtained from the Institute of Museum and Library Services to support a three‐year project on cryobanking, and to provide smaller grants to 10 other botanical gardens for one‐year projects on either (1) seed behavior studies or (2) the development of protocols for in vitro propagation or cryopreservation.

**Results:**

Nine of the partner gardens worked on 19 species (one was unable to continue due to the COVID‐19 pandemic), while the larger project focused on 14 species. A point system was developed for tasks accomplished, and the average costs per point of the larger and smaller projects were similar. Labor accounted for half the costs. Projects focused on species in the Asteraceae and Orchidaceae had lower costs per point than other species.

**Discussion:**

Both large and small projects can contribute to a strategy for exceptional plant conservation for similar costs. Prioritizing species with lower costs could help advance the field while allowing time for work on more difficult species to develop.

Exceptional plant species are those that cannot be conserved ex situ using conventional seed‐banking methods, instead requiring conservation as living collections or cryo‐collections, i.e., seeds or tissues stored in liquid nitrogen (LN) in cryobanks (Pence et al., 2022a). It is estimated that exceptional species number in the tens of thousands (Wyse and Dickie, 2017; Colville and Pritchard, 2019) and, as such, present a distinct challenge to the plant conservation community. While some groups of taxa are being organized into coordinated living collections, or metacollections, in botanical gardens (Fant et al., 2016; Westwood et al., 2021), limitations of space and resources mean the majority of threatened exceptional taxa will likely require LN storage, a method that has been shown to be effective for a variety of tissues from a wide range of species (Reed, 2008; Pence et al., 2020; Walters and Pence, 2020). Depending on the type of exceptionality, cryopreserved propagules can include seeds, embryos, dormant buds, or in vitro tissue cultures of shoot tips or somatic embryos.

Implementing cryostorage as a method for exceptional species conservation will require the harnessing of expertise and infrastructure for research in various areas, including seed biology, to determine whether seed behavior is exceptional; in vitro biology, to recover cryopreserved embryos or generate in vitro shoot tips or somatic embryos for banking; and cryopreservation. While the costs for these approaches will be greater than conventional seed banking, evaluating the actual costs and required resources is difficult because of the different types of exceptionality, the wide variation in species responses to these methods, and the resulting variation in the time and resources needed to tailor protocols to individual species. Understanding these costs is nevertheless critical for developing meaningful and workable strategies to meet the challenges of conserving these species.

Botanical gardens are well positioned for work in species conservation. While historically botanical gardens focused on taxonomy and plant discovery, the development of the field of conservation biology and the rise in threats to plant biodiversity worldwide have resulted in a more recent shift in focus to conservation science and research for many gardens (Smith, 2019). With an estimated 3269 botanical gardens and arboreta worldwide (Mounce et al., 2017), many institutions have begun to utilize their unique knowledge of plant propagation and access to collections in efforts to further our basic understanding of plant biology, in situ conservation, restoration and reintroduction, and public outreach and education, as well as to influence public policy (BGCI, 2022a).

The United Nations' Convention on Biological Diversity's Global Strategy for Plant Conservation Target 8 stipulated that at least 75% of threatened plant species be maintained in ex situ collections (Convention on Biological Diversity, 2012). Botanic gardens significantly contribute to achieving this target, particularly through their development of significant living collections and seed banks, as well as research programs focused on threatened species (Smith and Pence, 2017; Liu et al., 2020; Westwood et al., 2021). Many may also have relevant infrastructure and expertise available, although it may not currently be directed at exceptional species conservation (Havens et al., 2006). Finding ways to engage existing expertise and infrastructure within botanical gardens would increase the efficiency of any strategies developed for exceptional plants. A recent analysis indicated that very few known exceptional species are currently conserved in vitro or in cryopreserved collections (Pence et al., 2022b).

Infrastructure, training, and funding have long been identified as major impediments to the expansion of conservation research in botanical gardens, but there has been little study into the required resources or most efficient methods for increasing capacity (Havens et al., 2006). To address this, the Cincinnati Zoo & Botanical Garden's Center for Conservation and Research of Endangered Wildlife (CREW) received funding from the Institute of Museum and Library Services (IMLS) to develop cryopreservation protocols for a group of exceptional species from Hawai'i initiated into culture at the Lyon Arboretum (University of Hawai'i, Honolulu, Hawai'i, USA), as well as to provide small, one‐year grants to 10 U.S. botanical gardens to begin or continue the evaluation of seed behavior or protocol development for one or more exceptional species. Projects ranged from testing whether seeds were short lived using accelerated aging to developing tissue culture and/or cryopreservation protocols for confirmed exceptional species. The recorded metrics included costs, time, outcomes, and challenges. The structure of this exercise provided an opportunity to compare two approaches: a major investment into one institution and project, and the dissemination of small seed grants to multiple institutions. The lessons learned from these projects lend insight into the needs of conservation practitioners and approaches that might be used to develop effective strategies for conserving exceptional plant species.

## METHODS

### Grant funding and partner gardens

A grant from the IMLS was received by the Cincinnati Zoo & Botanical Garden to develop cryopreservation protocols for several species that had been initiated into in vitro culture in the Micropropagation Lab at the Lyon Arboretum and to bank multiple genotypes of these species in LN in CREW's CryoBioBank. In addition, funds were provided for projects in 10 partner gardens. These gardens were invited to present proposals for projects involving exceptional plant species, either (1) evaluating seed behavior to identify exceptional status; (2) developing an in vitro propagation protocol, which would be needed to provide tissues for, or recovery from, cryopreservation; or (3) developing a cryopreservation procedure for either seeds or tissues. The partners were then each awarded US$5000 to undertake the project over the course of a year, to be used per the grantee's discretion. The 10 gardens were located across the United States and included: Atlanta Botanical Garden (Atlanta, Georgia), Chicago Botanic Garden (Glencoe, Illinois), Denver Botanic Gardens (Denver, Colorado), Fairchild Tropical Botanic Garden (Coral Gables, Florida), Henry Doorly Zoo (Omaha, Nebraska), The Huntington Art Museum, Library, and Botanical Gardens (San Marino, California), Longwood Gardens (Kennett Square, Pennsylvania), Lyon Arboretum (Honolulu, Hawai'i), Missouri Botanical Garden (St. Louis, Missouri), and San Diego Zoo Wildlife Alliance (San Diego, California).

### Data collection and analysis

At the conclusion of the grant period, each garden was asked to respond to a survey regarding their species of concern, the methods used over the course of the project, detailed cost breakdowns (including both cost breakdowns within the $5000 seed grant and costs incurred beyond the grant; Appendix S1), staff labor hours, outcomes, and challenges encountered. Data were compiled across institutions and analyzed using R version 4.1.0 (R Core Team, 2021). Graphics were generated using the R package *ggplot2* (Wickham, 2016).

### Outcome analysis

In order to elucidate the impacts of varying levels of funding and resource allocation, outcomes were classified according to the three areas of work and assigned points as described in Box 1. Points were assigned for developing protocols contributing to these outcomes. Because the development of in vitro protocols involves several types of research, initiation/multiplication, in vitro rooting or somatic embryo germination, and acclimatization, these were considered separately. If a protocol was developed, this was assigned one point. A half point was assigned for research that increased knowledge but did not yet result in a finished protocol. In addition, each target exceptional species genotype (for tissues) or maternal line (for seeds) banked in a cryobank was assigned a half point. The costs per point were assessed using the total costs for each project.

Box 1 **Points assigned for steps in identifying exceptional plant status through seed behavior analysis, developing protocols for preparing in vitro tissues and recovering plants, and developing cryopreservation protocols and implementing the protocols for long‐term cryo‐storage.**

**Table:** 

**I. Identify**	Classify exceptional status (1)		
**II. Develop in vitro protocols (if needed)**	A. Initiate shoot/somatic embryo culture and multiply (1); or germinate orchid seeds (1)	B. Reconstitute plants (root in vitro shoots or germinate somatic or zygotic embryos in vitro) (1)	C. Acclimatize (1)
**III. Cryopreserve**	A. Develop cryopreservation protocol for seeds, embryos, dormant buds, in vitro tissues (1)	B. Implement cryopreservation protocol for banking seed lot or genotype (0.5)

## RESULTS

### Total costs

Nine of the 10 partner gardens completed projects; one was unable to finish because of the COVID‐19 pandemic. The nine gardens used a total of $155,650 to improve ex situ conservation methods for 19 different target exceptional plant species. Five of the nine institutions used funds beyond the $5000 seed grant, with the additional funds pulled primarily from their institutional resources, although one garden included funds from the Center for Plant Conservation and another from private donations. One garden used the IMLS seed money as leverage to obtain much larger funding for the project that allowed for the hiring of a temporary full‐time staff member. Because these funds were then combined with the IMLS seed money, this garden (Garden 9) is considered separately in our analysis.

**Figure 1:**
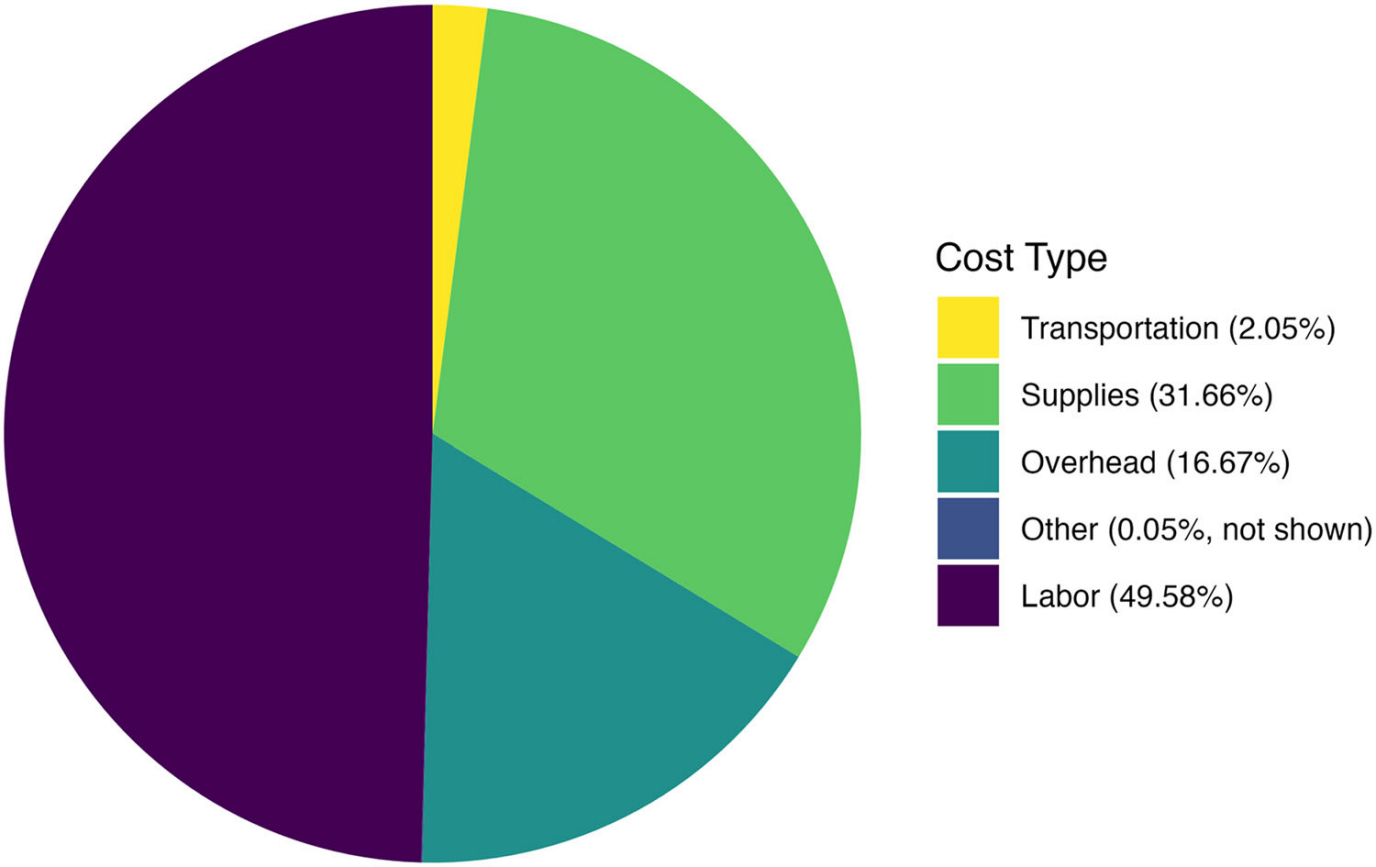
Percentage of total costs by cost type. Costs are displayed as percentages of the total costs, including any costs incurred beyond the initial seed grant.

Seven of the eight partners provided a breakdown of costs, of which nearly half were allocated toward labor (49.58%) with the next largest proportion spent on supplies (31.66%; Figure 1). All nine institutions provided the hours of labor dedicated to the project. Gardens 1–8 contributed 2536 h, while the addition of a full‐time position for Garden 9 increased the total to 5396 h for the entire group.

### Taxonomy of studied species

**Figure 2:**
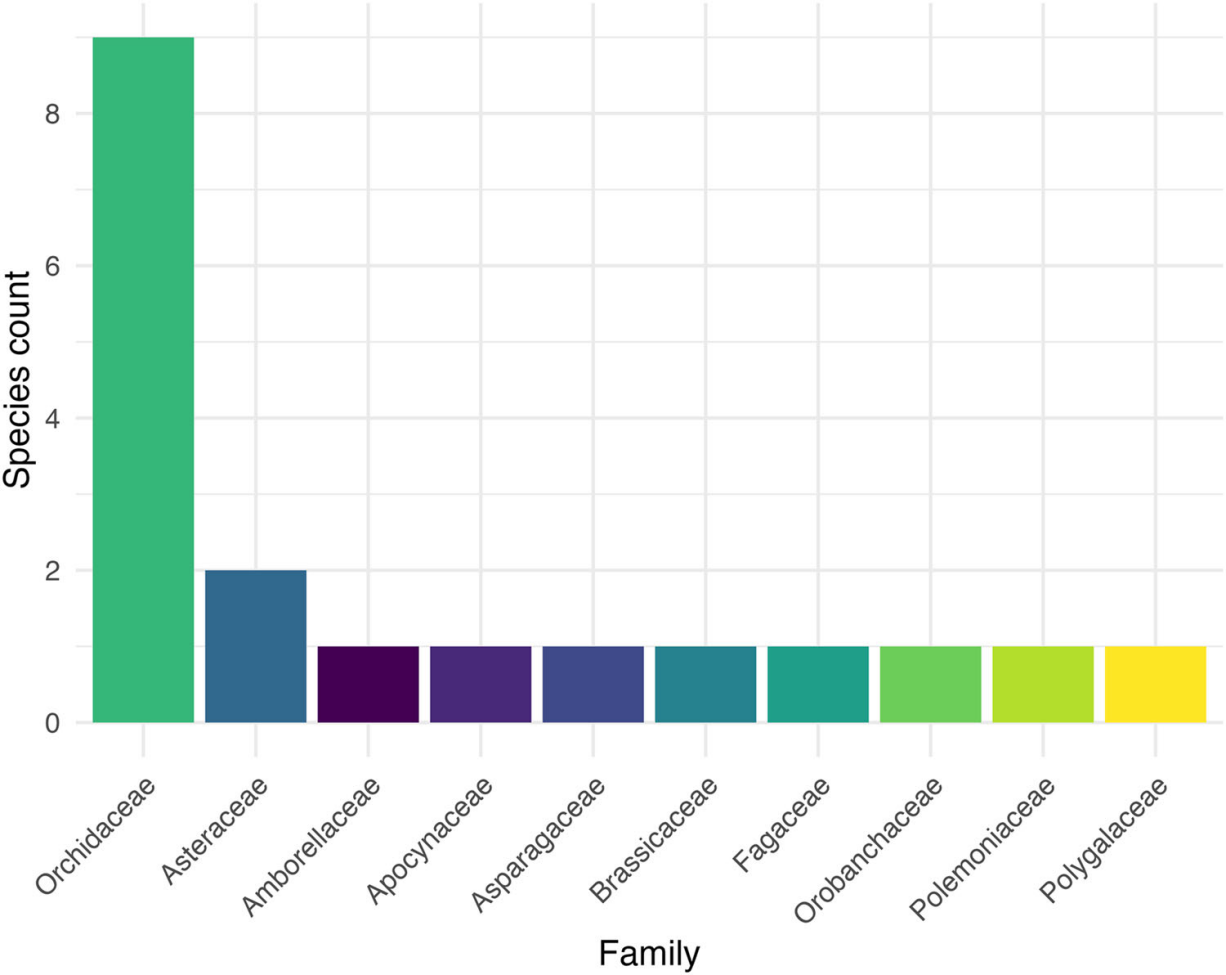
Total number of species in each family studied by partner gardens.

The variety of species studied was wide, with nine of the 19 target species in the Orchidaceae family, two species in the Asteraceae, and the remaining eight species from a range of plant families (Amborellaceae, Apocynaceae, Asparagaceae, Brassicaceae, Fagaceae, Orobanchaceae, Polemoniaceae, and Polygalaceae; Figure 2). According to the NatureServe (2021) Global (G) rankings, of the 19 species, three were considered to have a G2 (imperiled) status, seven were G3 (vulnerable), five were G4 (apparently secure), one was G5 (secure), and three were not assessed.

### Challenges identified

Most gardens (5/9) cited propagule acquisition as a challenge in working with their species, followed by issues of contamination (4/9) and a lack of information about species (4/9). Two gardens cited the COVID‐19 pandemic as a challenge. Other challenges identified included a difficulty identifying potential partnerships, invasive species, lack of funding, natural disasters, population dynamics, slow propagule growth, tissue browning, small population size, and training.

### Outcomes achieved

While only one‐third (3/9) of surveyed gardens stated that the grant increased the work they did on exceptional species, all gardens expressed an intention to continue working with exceptional species in general. The gardens collaborated with a variety of other institutions on the funded projects, including one additional botanical garden; a university; a high school; and a variety of local, state, and federal natural resource agencies.

### Outcome analysis

The number of outcome points achieved over the course of the project per partner garden ranged from 0.5 to 6. The costs per point ranged from $1716 to $10,277, with an average cost per point of $3517. The project that had obtained substantial additional funding for a full‐time position had a cost per point of $43,530. The overarching grant project managed by CREW was included as a comparison of differences in funding structures. CREW's project involved developing in vitro propagation and cryopreservation protocols and banking samples for 14 endangered exceptional Hawaiian species that had been initiated into culture at the Lyon Arboretum. Grant funding for the project at CREW was $208,800 over three years, with 7961 documented staff hours and 39 total outcome points achieved over the course of the project, resulting in an average cost per point of $5354.

## DISCUSSION

This study provided a real‐world exercise for evaluating the costs and resources needed for the ex situ conservation of exceptional plant species, specifically for those approaches focused on identifying exceptional behavior in seeds, initiating in vitro cultures, and cryopreserving seeds or tissues. This was done by funding smaller projects dealing with 1–4 species each, as well as a larger project focused on 14 species. Participants in the smaller projects were allowed to work on a project of their choosing, and thus the projects differed widely in the types of protocols studied and the methods used (Seglias, 2022; Zale et al., 2022). This situation reflects the variety of exceptional species requiring attention, the variety of challenges they pose, and the differences in laboratory approaches and expertise, although it also presents a challenge in comparing projects. The seed grant projects were compared with the investment made into one lab with a full‐time staff person over three years, who worked to adapt cultures initiated in another lab to cryopreservation protocols and to bank multiple genotypes of those cultures. Using the point system developed for this project to attempt to measure the effort invested into the different types of activities, the costs per point were similar for both types of projects; the multiple smaller grants had an average cost per point of $3517, compared with $5354 for the larger project dealing with multiple species.

There have been other estimates of the costs of initiating and maintaining in vitro collections and of cryobanking plant seeds and tissues, particularly for crop species, including coffee (*Coffea* L. spp.) (Dulloo et al., 2009), temperate fruit trees and cassava (*Manihot esculenta* Crantz) (Reed et al., 2004), garlic (*Allium sativum* L.) (Keller et al., 2013), banana (*Musa acuminata* Colla) (Panis et al., 2020), and potato (*Solanum tuberosum* L.) (Keller et al., 2013). It is often difficult to compare these costs because of the differences in explant type, differences in the number of subcultures necessary to obtain tissues for cryobanking, and previous work on the species (Pence, 2011; Keller et al., 2013). Labor is often cited as the largest portion of the total costs (Keller et al., 2013), which was also true in our study. In addition, most previous analyses have been performed on economically important species about which much is known, and for which there are often established in vitro and cryopreservation protocols. A 2011 cost estimate for the development of a protocol for cryopreserving the embryos or tissues of wild threatened species was about $2000 (Pence, 2011), slightly lower than the averages obtained from the tracking performed in the current project, and generally higher than those given for cryopreserving crop species.

The development of an in vitro or cryopreservation protocol for a new species is unpredictable in terms of the investment in time and resources that will be required. Our point system deals with the individual steps in a complete protocol, rather than the finalized method, reflecting the realities of research, which deals with each of these steps in succession. Each step can present different challenges, which vary by species. Some species present very little issue; for example, one project dealt with developing an in vitro protocol for a threatened species within the Asteraceae, *Cirsium hillii* Fernald, resulting in a full in vitro propagation procedure, including initiation/multiplication, rooting, and acclimatization, for one of the lowest costs in this study, $1716 per point. This likely reflects the fact that many Asteraceae species have been successfully propagated in vitro (Abraham and Thomas, 2016), suggesting that a number of these species adapt well to in vitro manipulation. However, although the initiation of cultures of several *Quercus* L. species has been reported (Ballesteros and Pritchard, 2020), oaks are known to be difficult to initiate in vitro and the rates of initiation are often low (Kramer and Pence, 2012; Brennan et al., 2017; Winkeljohn et al., 2022). This is reflected in the higher cost per point ($43,530) in a project dealing with *Q. dumosa* Nutt.

The differential response to in vitro propagation or cryopreservation was observed with other species, resulting in the differences in costs for these studies. Orchids are another group for which there has been a significant amount of in vitro research reported in the literature (Chugh et al., 2009). The three projects focused on orchids (excluding the project used to leverage further funds) ranked lower in cost, ranging from $1579 to less than $3600 per point (the latter project on orchids also included a non‐orchid and the costs were not separated). These projects involved testing the effects of media on in vitro germination (Zale et al., 2022), as well as some testing of the seeds for tolerance to cryopreservation and banking, and these types of projects benefited from both the large volume of literature available on orchid seed germination in vitro and the large number of seeds available for experimental treatments (Kauth et al., 2008; Jolman et al., 2022). In addition, one garden working with orchid seeds noted that, although much of the cryopreservation work was dependent on full‐time skilled labor, some components, such as curation, viability testing, and data collection, could be done by trained volunteers and students, which helped to offset costs and scale up the work. On the other hand, of the four projects exceeding $5000 (the amount provided by the IMLS grant for the project) per point, one required the purchase of equipment for the project (Seglias, 2022) and three dealt with species that appear to be particularly difficult to initiate into culture: *Alyxia stellata* (J. R. Forst. & G. Forst.) Roem. & Schult. was challenging to sterilize, *Polygala lewtonii* Small had limited material with reduced viability, and *Q. dumosa* showed severe browning in culture.

One additional benefit to the structure of this project was the built‐in element of collaboration between the participating institutions. By coming together for a meeting and discussion at the onset of the project, researchers were able to pool knowledge resources on best practices before undertaking their individual projects. A similar meeting at the conclusion of the project to share successes and challenges led to the analysis of costs and necessary resources presented here. This model of open communication and collaboration between practitioners in exceptional plant conservation could increase efficiency in exceptional plant research by building on shared knowledge and reducing duplication efforts. For effective conservation in any species, open communication and collaboration is key.

Unsurprisingly, roughly half of all costs went toward labor, with more than one‐third going toward supplies, and the rest to other expenses. This demonstrates the need for increased funding and training in the field for exceptional plant conservation specialists. Most of the participating gardens already had some infrastructure for their exceptional plant conservation projects, from seed‐testing facilities, to tissue‐culture labs, to a source of LN; however, using additional funds, one lab purchased equipment for seed work for this project, which it is continuing to use (Seglias, 2022). The supply costs would necessarily increase for a lab just starting in exceptional species research, as it would have to build the necessary infrastructure for these specialized techniques. Of the over 3758 botanical gardens providing information to Botanic Gardens Conservation International (BGCI)'s Garden Search, 177 indicate that they have tissue culture/micropropagation facilities (BGCI, 2022b). For such labs, labor and supplies will be the primary costs.

This study demonstrates that a nominal investment of funds can help increase primary research and method development for exceptional species and their effective conservation, even if full protocols are not achieved immediately. With a relatively low investment of $5000 per partner garden ($45,000 total), the ex situ conservation needs of 19 exceptional species were furthered, in some cases by leveraging or obtaining additional funds. While one goal of this project was to facilitate research on exceptional plants, an overarching goal was to provide additional supporting information to guide the development of strategies for exceptional plant conservation. To that end, several points can be made from this work, as outlined below.

### Expand networking and information

Participants were asked to discuss the challenges they encountered over the course of the projects, and two major categories emerged: a lack of information on target species and a lack of networking and partnerships. These are two of the three major challenges to exceptional plant conservation that were identified in 2013 in the Dunedin Statement of Need (https://cincinnatizoo.org/system/assets/uploads/2019/02/Dunedin-statement-of-need.pdf). Propagule acquisition was the specific challenge most commonly mentioned by the collaborating group in the present project, which could potentially be alleviated by increased partnerships and collaboration, particularly with field botanists. Avenues for networking need to be increased; however, the BGCI's Global Conservation Consortia (https://www.globalconservationconsortia.org/), the Exceptional Plant Conservation Network (http://cincinnatizoo.org/epcn), and regional conservation networks such as the Center for Plant Conservation (http://saveplants.org) and the Australian Network for Plant Conservation (https://www.anpc.asn.au/), among others, provide platforms for such networking and should be utilized more fully for facilitating exceptional plant research and increasing training opportunities.

A lack of information underlies a number of other challenges that were identified, specifically contamination, lack of basic research on the target species, slow propagule growth, and tissue browning. These are common challenges when dealing with wild species (Benson, 2000; Dong et al., 2016; Quambusch and Winkelmann, 2018; Volk et al., 2022) and underscore the need for a greater understanding of the basic biology of a species in areas of seed behavior, in vitro growth, and responses to cryopreservation. While most protocols in the literature have been developed for species of economic importance, not all of these methods are easily transferred to wild species, particularly those unrelated to crop species and about which little is known. Facilitating more research in protocol development, particularly for rare taxa, can add to the body of information to support future work.

### Leverage species that are most adaptable to conservation methods

The differences in the outcomes for the species under study in this project suggest that investments into congeners of better‐known species in families such as the Asteraceae and Orchidaceae may provide the most rapid return on investment, as work progresses more slowly on more poorly understood or “difficult” species. In a recent analysis, there were 802 threatened congeners of exceptional species in these two families, and these should be prioritized for research (Pence et al., 2022b). Additionally, testing for seed longevity and work with orchid seeds will likely require less investment per species than taxa such as *Quercus* species or other trees. While work on the latter is critical, developing a strategy to target efforts to those species that can most quickly benefit can help in building infrastructure and networks that can then deal with the more challenging taxa.

### Maximize infrastructure and expertise

This project compared outcomes from small seed grants and a larger investment in one lab and highlighted the benefits of each. Small grants may be particularly useful for evaluating seed behaviors and developing protocols for threatened under‐studied species, particularly if students, volunteers, and academic partners can be enlisted, as they were in some of these projects. Our project also brought these institutions together to discuss their projects, challenges, and results, which promoted networking within the group. For institutions with the resources to allocate dedicated staff specifically to exceptional plant research, larger projects might center on developing protocols to scale to bank multiple species and genotypes. Such projects will likely be collaborative as well, as was the case with the collaboration between CREW and the Lyon Arboretum. The work at CREW was based on a large body of work at the Lyon Arboretum, which provided the cultures that served as the basis for culture multiplication, cryopreservation, and banking. Thus, these outcomes all point to a model for exceptional plant conservation that leverages the infrastructure and various areas of expertise of both small and larger programs in botanical gardens, perhaps developing protocols in the former and undertaking more large‐scale propagation and banking in the latter.

The efficiencies of conserving exceptional plants is a primary challenge and will not be equivalent to those of seed banking; however, projects such as this one can provide a basis for developing more efficient strategies for dealing with the conservation of the thousands of plant species that are predicted to be exceptional. This study illustrates that a relatively small investment can make a significant difference, and further investment into both large and small projects is needed to advance the science and the collaborations necessary to meet the challenge of exceptional plant conservation.

## AUTHOR CONTRIBUTIONS

M.P. and V.C.P. contributed results, analyzed data, and wrote the manuscript. B.B., A.S.C., E.E.D.C., J.L.D., C.E.E., R.F., J.J.L., C.P., J.F.R., A.E.S., N.S., P.J.Z., and J.Z. contributed results and comments to the manuscript. All authors approved the final version of the manuscript.

## Data Availability

The detailed cost and points breakdown for this study is available in the Supporting Information as Appendix S1.

## References

[aps311495-bib-0040] Abraham, J. , and T. D. Thomas . 2016. Recent advances in Asteraceae tissue culture. *In* M. Anis and N. Ahmad [eds.], Plant tissue culture: Propagation, conservation and crop improvement, 161–195. Springer, Singapore. 10.1007/978-981-10-1917-3_9

[aps311495-bib-0001] Ballesteros, D. , and H. W. Pritchard . 2020. The cryobiotechnology of oaks: An integration of approaches for the long‐term *ex situ* conservation of *Quercus* species. Forests 11: e1281. 10.3390/f11121281

[aps311495-bib-0002] Benson, E. E. 2000. In vitro plant recalcitrance: An introduction. In Vitro Cellular & Developmental Biology–Plant 36: 141–148.

[aps311495-bib-0003] BGCI . 2022a. About botanic gardens. Botanic Gardens Conservation International. Website: https://www.bgci.org/about/about-botanic-garden/ [accessed 13 January 2022].

[aps311495-bib-0004] BGCI . 2022b. GardenSearch. Botanic Gardens Conservation International. Website: https://tools.bgci.org/garden_advanced_search.php [accessed 14 January 2022].

[aps311495-bib-0005] Brennan, A. N. , V. C. Pence , M. D. Taylor , B. W. Trader , and M. Westwood . 2017. Tissue culture using mature material for the conservation of oaks. HortTechnology 27: 644–649. 10.21273/HORTTECH03801-17

[aps311495-bib-0039] Chugh, S. , S. Guha , and I. U. Rao . 2009. Micropropagation of orchids: A review on the potential of different explants. Scientia Horticulturae 122(4): 507–520. 10.1016/j.scienta.2009.07.016

[aps311495-bib-0006] Colville, L. , and H. W. Pritchard . 2019. Seed life span and food security. New Phytologist 224: 557–562. 10.1111/nph.16006 31225902

[aps311495-bib-0007] Convention on Biological Diversity . 2012. Global strategy for plant conservation: 2011–2020. Botanic Gardens Conservation International, Richmond, United Kingdom.

[aps311495-bib-0008] Dong, Y. S. , C. H. Fu , P. Su , X. Xu , J. Yuan , S. Wang , M. Zhang , et al. 2016. Mechanisms and effective control of physiological browning phenomena in plant cell cultures. Physiologia Plantarum 156: 13–28. 10.1111/ppl.12382 26333689

[aps311495-bib-0009] Dulloo, M. E. , A. W. Ebert , S. Dussert , E. Gotor , C. Astorga , N. Vasquez , J. J. Rakotomalala , et al. 2009. Cost efficiency of cryopreservation as a long‐term conservation method for coffee genetic resources. Crop Science 49: 2123–2138. 10.2135/cropsci2008.12.0736

[aps311495-bib-0010] Fant, J. B. , K. Havens , A. T. Kramer , S. K. Walsh , T. Callicrate , R. C. Lacy , M. Maunder , et al. 2016. What to do when we can't bank on seeds: What botanic gardens can learn from the zoo community about conserving plants in living collections. American Journal of Botany 103: 1541–1543. 27578628 10.3732/ajb.1600247

[aps311495-bib-0011] Havens, K. , P. Vitt , M. Maunder , E. O. Guerrant , and K. Dixon . 2006. *Ex situ* plant conservation and beyond. BioScience 56: 525–531. 10.1641/0006-3568(2006)56[525:ESPCAB]2.0.CO;2

[aps311495-bib-0012] Jolman, D. , M. I. Batalla , A. Hungerford , P. Norwood , N. Tait , and L. E. Wallace . 2022. The challenges of growing orchids from seeds for conservation: An assessment of asymbiotic techniques. Applications in Plant Sciences 10(5): e11496. 10.1002/aps3.11496 PMC9575117 36258786

[aps311495-bib-0013] Kauth, P. J. , D. Dutra , T. R. Johnson , S. L. Stewart , M. E. Kane , and W. Vendrame . 2008. Techniques and applications of in vitro orchid seed germination. *In* J. A. Teixeira da Silva [ed.], Floriculture, ornamental and plant biotechnology: Advances and topical issues, vol. 5, 375–391. Global Science Books, Middlesex, United Kingdom.

[aps311495-bib-0014] Keller, E. R. J. , C. D. Zanke , A. Senula , A. Breuing , B. Hardeweg , and T. Winkelmann . 2013. Comparing costs for different conservation strategies of garlic (*Allium sativum* L.) germplasm in genebanks. Genetic Resources Crop Evolution 60: 913–926. 10.1007/s10722-012-9888-5

[aps311495-bib-0015] Kramer, A. , and V. Pence . 2012. The challenges of *ex situ* conservation for threatened oaks. International Oak Journal 23: 91–107.

[aps311495-bib-0016] Liu, U. , T. A. Cossu , R. M. Davies , F. Forest , J. B. Dickie , and E. Bremen . 2020. Conserving orthodox seeds of globally threatened plants *ex situ* in the Millennium Seed Bank, Royal Botanic Gardens, Kew, UK: The status of seed collections. Biodiversity Conservation 29: 2901–2949. 10.1007/s10531-020-02005-6

[aps311495-bib-0017] Mounce, R. , P. Smith , and S. Brockington . 2017. *Ex situ* conservation of plant diversity in the world's botanic gardens. Nature Plants 3: 795–802. 10.1038/s41477-017-0019-3 28947807

[aps311495-bib-0018] NatureServe . 2021. NatureServe Network Biodiversity Location Data accessed through NatureServe Explorer [web application]. NatureServe, Arlington, Virginia. Website: https://explorer.natureserve.org/ [accessed 1 October 2021].

[aps311495-bib-0019] Panis, B. , M. Nagel , and I. V. den Houwe . 2020. Challenges and prospects for the conservation of crop genetic resources in field genebanks, in in vitro collections and/or in liquid nitrogen. Plants 9: 1634. 10.3390/plants9121634 PMC7761154 33255385

[aps311495-bib-0020] Pence, V. C. 2011. Evaluating costs for the in vitro propagation and preservation of endangered plants. In Vitro Cellular and Developmental Biology ‐ Plant 47: 176–187.

[aps311495-bib-0021] Pence, V. C. , D. Ballesteros , C. Walters , B. M. Reed , M. Philpott , K. W. Dixon , H. W. Pritchard , et al. 2020. Cryobiotechnologies: Tools for expanding long‐term *ex situ* conservation to all plant species. Biological Conservation 250: 108736. 10.1016/j.biocon.2020.108736

[aps311495-bib-0022] Pence, V. C. , A. Meyer , J. Linsky , J. Gratzfeld , H. W. Pritchard , M. Westwood , and E. B. Bruns . 2022a. Defining exceptional species: A conceptual framework to expand and advance *ex situ* conservation of plant diversity beyond conventional seed banking. Biological Conservation 266: 109440. 10.1016/j.biocon.2021.109440

[aps311495-bib-0023] Pence, V. C. , E. B. Bruns , A. Meyer , H. W. Pritchard , M. Westwood , J. Linsky , J. Gratzfeld , et al. 2022b. Gap analysis of exceptional species: Using a global list of exceptional status to inform strategic conservation action. Biological Conservation 266: 109439.

[aps311495-bib-0024] Quambusch, M. , and T. Winkelmann . 2018. Bacterial endophytes in plant tissue culture: Mode of action, detection, and control. *In* V. M. Loyola‐Vargas and N. Ochoa‐Alejo [eds.], Plant cell culture protocols, 69–88. Methods in Molecular Biology, 1815. Humana Press, New York, New York, USA. 10.1007/978-1-4939-8594-4_4 29981114

[aps311495-bib-0025] R Core Team . 2021. R: A language and environment for statistical computing. R Foundation for Statistical Computing, Vienna Austria. Website: https://www.R-project.org/ [accessed 25 January 2022].

[aps311495-bib-0026] Reed, B. M. [ed.] 2008. Plant cryopreservation: A practical guide. Springer Science+Business Media, New York, New York, USA.

[aps311495-bib-0027] Reed, B. M. , F. Engelmann , M. E. Dulloo , and J. M. M. Engels . 2004. Technical guidelines for the management of field and in vitro germplasm collections. IPGRI Handbook for Genebanks, No. 7. International Plant Genetic Resources Institute, Rome, Italy.

[aps311495-bib-0028] Seglias, A. E. 2022. Can alpine plant species “bank” on conservation?: Using artificial aging to understand seed longevity. Applications in Plant Sciences 10(5): e11493. 10.1002/aps3.11493 PMC9575086 36258790

[aps311495-bib-0029] Smith, P. 2019. The challenge for botanic garden science. Plants, People, Planet 1: 38–43. 10.1002/ppp3.10

[aps311495-bib-0030] Smith, P. , and V. C. Pence . 2017. The role of botanic gardens in *ex situ* conservation. *In* S. Blackmore and S. Oldfield [eds.], Plant conservation science and practice: The role of botanic gardens. Cambridge University Press, Cambridge, United Kingdom.

[aps311495-bib-0031] Volk, G. M. , R. Bonnart , A. C. Araùjo de Oliveira , and A. D. Henk . 2022. Minimizing the deleterious effects of endophytes in plant shoot tip cryopreservation. Applications in Plant Sciences 10(5): e11489. 10.1002/aps3.11489 PMC9575093 36258787

[aps311495-bib-0032] Walters, C. , and V. C. Pence . 2020. The unique role of seed banking and cryobiotechnologies in plant conservation. Plants, People, Planet 3: 83–91. 10.1002/ppp3.10121

[aps311495-bib-0033] Westwood, M. , N. Cavender , A. Meyer , and P. Smith . 2021. Botanic garden solutions to the plant extinction crisis. Plants, People, Planet 3: 22–32. 10.1002/ppp3.10134

[aps311495-bib-0034] Wickham, H. 2016. ggplot2: Elegant graphics for data analysis. Springer‐Verlag, New York, New York, USA.

[aps311495-bib-0035] Winkeljohn, M. , V. C. Pence , and T. M. Culley . 2022. Improving culture initiation of mature oak shoots through use of silver thiosulfate. Applications in Plant Sciences 10(5): e11497. 10.1002/aps3.11497 PMC9575081 36258789

[aps311495-bib-0036] Wyse, S. V. , and J. B. Dickie . 2017. Predicting the global incidence of seed desiccation sensitivity. Journal of Ecology 105: 1082–1093. 10.1111/1365-2745.12725

[aps311495-bib-0037] Zale, P. J. , A. Clayton , J. Nix , and M. Taylor . 2022. Asymbiotic in vitro seed germination, in vitro seedling development, and ex vitro acclimatization of *Spiranthes* . Applications in Plant Sciences 10(5): e11494. 10.1002/aps3.11494 PMC9575095 36258788

